# Photobiomodulation therapy

**DOI:** 10.1016/j.jfscie.2025.100045

**Published:** 2025-03-26

**Authors:** Praveen R. Arany

**Affiliations:** Departments of Oral Biology, Surgery, and Biomedical Engineering, School of Dental Medicine, Jacobs School of Medicine and Biomedical Sciences, School of Engineering and Applied Sciences, University at Buffalo, The State University of New York, Buffalo, NY

**Keywords:** Photobiomodulation, low-level light/laser therapy, TGF-β1

## Abstract

**Objectives:**

The use of low doses of photons (light) in biological tissues to modulate (inhibit or stimulate) responses is termed photobiomodulation (PBM). This nonthermal, noninvasive, and nonsurgical light treatment has been reported to reduce pain or inflammation, modulate immune responses, and promote tissue healing and regeneration. These fundamental pathophysiological responses underlie several oral and dental diseases, highlighting the broad scope of PBM interventions such as alleviating pain, discomfort, and swelling postsurgical procedures, including third-molar extractions, managing oncotherapy-associated mucositis and temporomandibular joint disorders, and promoting accelerated orthodontic tooth movements and implant osseointegration.

**Search Strategy, Citation Sources, and Data Elements:**

This narrative review provides the state-of-the-art in the PBM field, including history, terminology, mechanisms, devices, safety, regulations, and policy. The primary emphasis of this work is to outline the advances in mechanistic insights and clinical dosing paradigms that enable the safe and effective use of this therapy.

**Overall Conclusions:**

The importance of fundamental PBM education and training concepts focusing on light-tissue interactions, target tissue composition, evoked therapeutic biological responses, clinical diagnosis, and rationalized dose prescriptions is emphasized. Furthermore, several issues and logistical concerns should be addressed to enable the routine use of this innovative nonpharmacological treatment. A succinct version of this article is available as the American Dental Association Technical Report no. 189 Standards Committee on Dental Products Working Group 6.58.

## Graphical abstract

**Figure:**
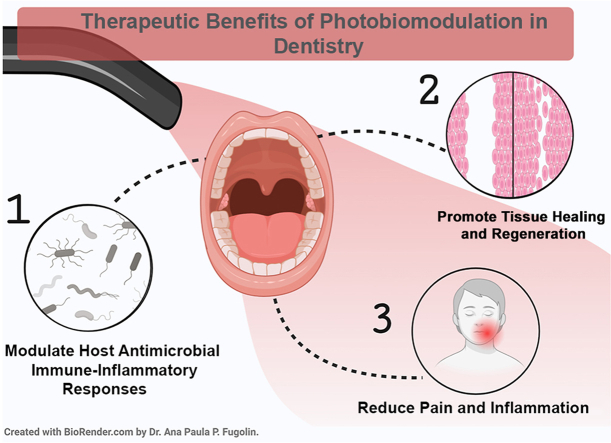

## Introduction

The beneficial effects of light were documented in the earliest records of civilization, such as the use of sunlight for various human ailments, from depression to general wellness. The development of optics and biophotonics, especially lasers, in the late 1960s has enabled the availability of a large number of light treatment devices. The invention of the clinical high-power laser was a substantial milestone in health care, revolutionizing medical and surgical fields such as dermatology, oncology, dentistry, plastic surgery, and ophthalmology. Several mainstream surgical applications are considered the standard of care, such as laser-assisted in situ keratomileusis for vision correction, vascular tumor resection, and skin photorejuvenation. The widespread availability of well-engineered light devices has enabled nonsurgical clinical treatment using a range of wavelengths and doses. Examples of well-established phototherapies include Psoralen plus ultraviolet A for psoriasis, blue-light incubators for neonatal jaundice, photopheresis for graft vs host disease, and narrow-band ultraviolet for psoriasis. Mester^1^, ^2^, ^3^ first observed the use of low-dose light treatments in the early 1960s as he explored the biological responses of the newly invented ruby laser. His initial rat studies reported improved wound healing and hair (fur) regrowth.^1^, ^2^, ^3^ Since then, the field has accrued evidence from controlled laboratory and human studies that have led, in several cases, to recommendations of low-dose light treatments as clinical practice guidelines. The broad applications of photobiomodulation (PBM) span various clinical specialties and cross the scope of practice, and its applications in dentistry have gained attention with rigorous clinical evidence.

There are several scientific organizations dedicated to promoting the science and benefits of PBM, such as the World Association for PhotobiomoduLation Therapy (WALT), North American Association for PhotobiomoduLation Therapy, the Multinational Association for Supportive Care in Cancer, the Academy of Laser Dentistry, Optica (formerly Optical Society for America), Society for Photonics and Optics Engineering, and Lasers Bio-Photonics Group of the International Association for Dental Research. The importance of PBM is heralded by its recommendation as a routine procedure in supportive cancer care, which further highlights the importance of oral health as a key aspect of overall general health. A detailed version of this article was accepted by the American Dental Association Technical Report no. 189 Standards Committee on Dental Products Working Group 6.58, which inspired this study. The US Food and Drug Administration (FDA) has also provided new device recommendations recognizing PBM as a discrete form of light treatment, which is a major advance in the field.^4^ These all represent a significant advance from PBM combined with conventional surgical and broader nonheating light treatments. This study provides a summary of the state of the field, acknowledging the progress and future avenues of a rapidly evolving and popular field.^5^

## Discrete Forms of Clinical Light Therapies Based on Their Evoked Biological Responses

**Figure 1:**
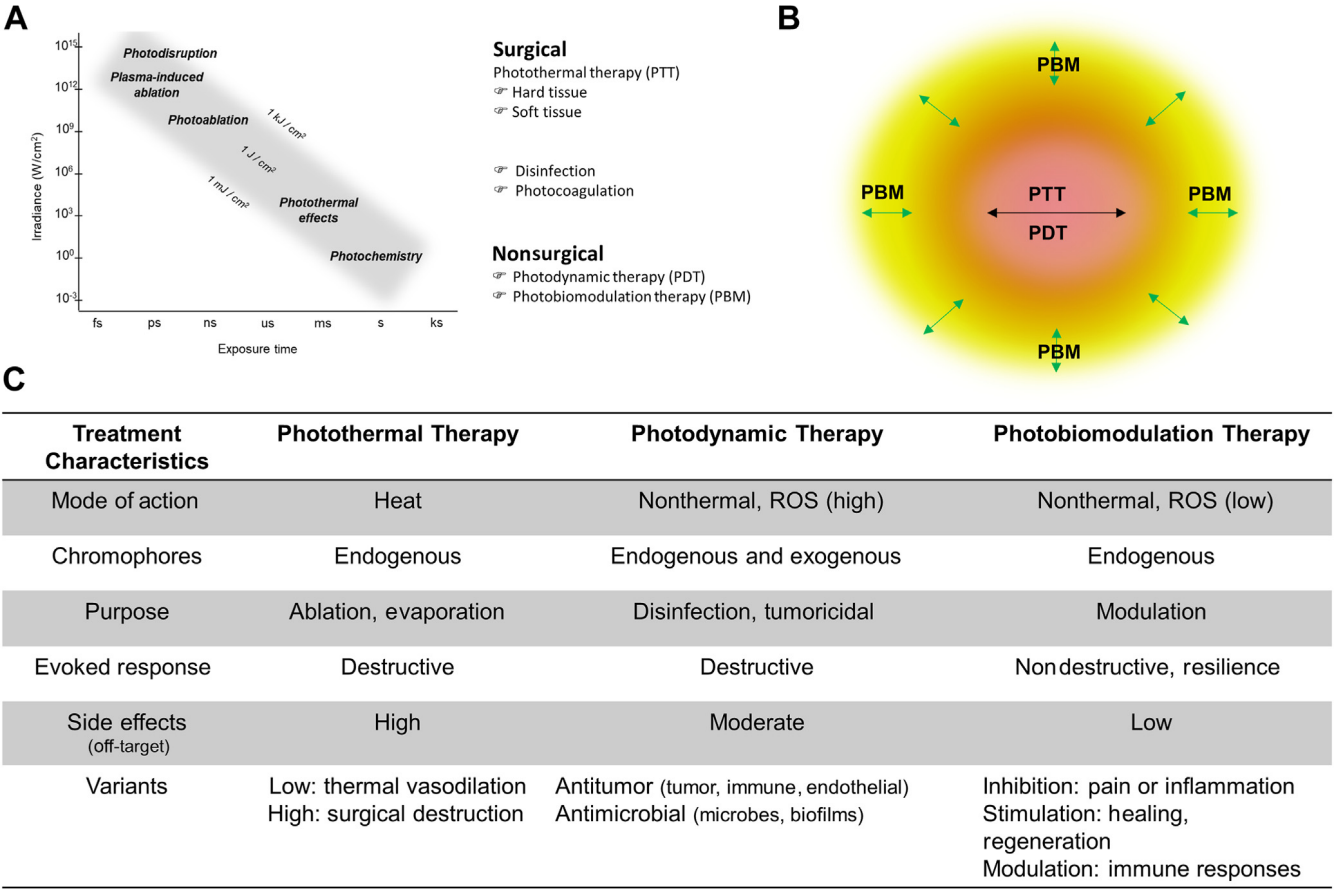
**A.** The light-biological tissue interaction is a dose-dependent response that can be directed explicitly to desirable clinical outcomes. **B.** The natural dispersion of light energy around higher dose applications, such as photothermal therapy (PTT) or photodynamic therapy (PDT), creates a subjacent zone of low-dose light-tissue interactions that is within the photobiomodulation (PBM) realm. **C.** Similarities and differences between PTT, PDT, and PBM that enable selective clinical use. There are specific clinical scenarios in which their rationalized biological responses can yield significant combinatorial use. fs: Femtosecond. ks: Kilosecond. ms: Millisecond. ns: Nanosecond. ps: Picosecond. ROS: Reactive oxygen species. s: Second.

There has been progress in our understanding of light-biological tissue interactions, enabling the delineation of discrete forms of light treatment approaches (Figure 1A).^6^, ^7^, ^8^ Rapid transfer of high energy into biological tissues using high-power lasers leads to instant disruption and evaporation.^9^, ^10^, ^11^ This photothermal therapy (PTT) forms the basis of surgical soft- and hard-tissue lasers and a thermal approach to polymicrobial biofilm disinfection. The latter is prone to inadvertent thermal responses in subjacent tissues and is often used for soft-tissue management (debridement) with primary disinfection. Among the advantages of surgical lasers compared with instruments are their precision and concurrent photocoagulation, generating a bloodless operative field that is particularly attractive for rich vascular orodental tissues. The often-reported reduced pain and inflammation in postlaser surgical processes compared with mechanical instrumentation has been attributed to reduced blood loss and minimal inadvertent damage (improved visualization of the operative field).^12^, ^13^, ^14^, ^15^ These are arguably within the lower energy zones that are within the PBM low-dose ranges (Figure 1B).^5^ There have been a few attempts at light dose distribution modeling for PBM that require careful extension to understand these surrounding light dose zones better.^16^, ^17^, ^18^, ^19^, ^20^

In contrast to the high-energy light interactions, lower amounts of light dose along with a chromophore resulting in a nonthermal, photochemical generation of reactive oxygen species (ROS) is termed photodynamic therapy (PDT). This technique uses both endogenous and exogenous chromophores (photosensitizers) to target microbes (antimicrobial photodynamic therapy [aPDT]) and tumor cells (antitumor photodynamic therapy [tPDT]). Absorption of a specific wavelength of light is enabled by chromophores derived from natural (eg, hemoglobin) or synthetic (eg, 5-aminolevulinic acid) biomolecules that generate ROS via type I (intermediate) or II (molecular oxygen) reactions. Independent of PTT and PDT responses, low doses of light energy can simulate or inhibit biological responses (ie, PBM). This approach generates low amounts of ROS that act on specific biological targets to modulate pathophysiological therapeutic responses. The most prominent differentiating feature between PBM and PDT is that the ultimate purpose of PDT is the irreversible destruction of its biological target.^7^ In contrast, PBM modulates biological responses that are transient and reversible but are primarily a nondestructive approach. Although PTT involves a range of elevated tissue temperatures from coagulation at 85 °C, vaporization at 100 °C, and carbonization at 200 °C, PBM is inherently nonthermal, although elevation of tissue temperature up to 45 °C has been reported to remain within the therapeutic responses.^21^ An article reported the use of upconverting or downconverting intermediate chromophores to extend PBM treatment delivery to distant anatomic regions.^22^ This may be particularly relevant to orodental scenarios with limited accessibility in periapical or complex periodontal furcation regions. For clarity, these intermediate targets do not represent a primary site for light-energy conversion, as is evident from PDT type I or II reactions. Instead, these exogenous chromophores emit a more suitable wavelength for desirable PBM therapeutic responses and, therefore, should not be categorized as PDT.

The advent of PDT can be traced back to the seminal work by the Danish physician Niels Rydberg Finsen in the late 18th century, who used concentrated sunlight to treat lupus vulgaris of the skin and received the 1903 Nobel Prize in Physiology or Medicine.^23^ There has been interest in clinical oral and dental applications of PDT, including caries, intracanal disinfections, periodontitis, periimplantitis, and premalignant and malignant lesions.^24^, ^25^, ^26^, ^27^ Advances in the bioengineering fields have enabled the availability of many sophisticated chromophores, from new dyes to targeted nanoformulations, along with superior light sources.^28^, ^29^, ^30^, ^31^ PDT approaches can be broadly categorized as tPDT and aPDT. Among tPDT, increasing attention has been paid to discrete lineages within the tumor, such as the tumor cell itself, tumor endothelial cells, and tumor immune cells. The latter has gained renewed attention with the advent of cancer immunotherapies.^32^^,^^33^ The aPDT field has been popularized for its ability to target the growing list of antimicrobial-resistant species.^34^, ^35^, ^36^ The overall scope and potential impact of PDT in oral and dental diseases has garnered interest and is poised to affect the field positively.

The 3 approaches of PTT, PDT, and PBM have often been used in combination synergistically or inadvertently, especially in dentistry, in which they are expected to evoke complementary biological responses that are ultimately clinically beneficial (Figure 1C). One of the best examples is the use of multimodal laser treatments in clinical dentistry for periodontal disease, in which there are discrete effects of PTT and PDT on tissue debridement or curettage, and reducing biofilms evokes a partial immunogenic response.^6^ Moreover, PBM has been reported to modulate potent host immune responses directly.^8^ Hence, an improved understanding and rationalized application of a laser procedure with discrete device parameters and protocols can evoke a directed light-biological tissue interaction. The ultimate clinical outcomes could involve tissue curettage, debridement, disinfection, and biomodulation for discrete therapeutic clinical benefits.

## Terminology

A search outlined more than 300 different terms, including some of the most common ones, such as low-level light or laser treatments, cold lasers, infrared therapy, and red-light therapy. A consensus meeting in 2014 at Arlington, Virginia, of WALT and the North American Association for Photobiomodulation Therapy recommended using the term PBM as it describes the biological modulation process with photonic (light) energy. The National Library of Medicine has adopted this as a Medical Subject Heading term that describes PBM as the “use of non-ionizing photonic energy to generate non-thermal therapeutic benefits.”^9^ A more elaborate definition the field uses indicates that

PBM is a form of light therapy that utilizes nonionizing forms of light sources, including lasers, LEDs, and broadband light, in the visible and infrared spectrum. It is a nonthermal process involving endogenous chromophores eliciting photobiophysical (ie, linear and nonlinear) and photobiochemical events at various biological scales. This process results in beneficial therapeutic outcomes including, but not limited to, alleviating pain or inflammation, immunomodulation, and promoting tissue healing and regeneration.^9^

The term photobiomodulation therapy has been used colloquially, supposedly to parallel the use of the term PDT, which seems acceptable.

## Scope and Evidence for PBM in Oral, Dental, and Craniofacial Disease

**Figure 2:**
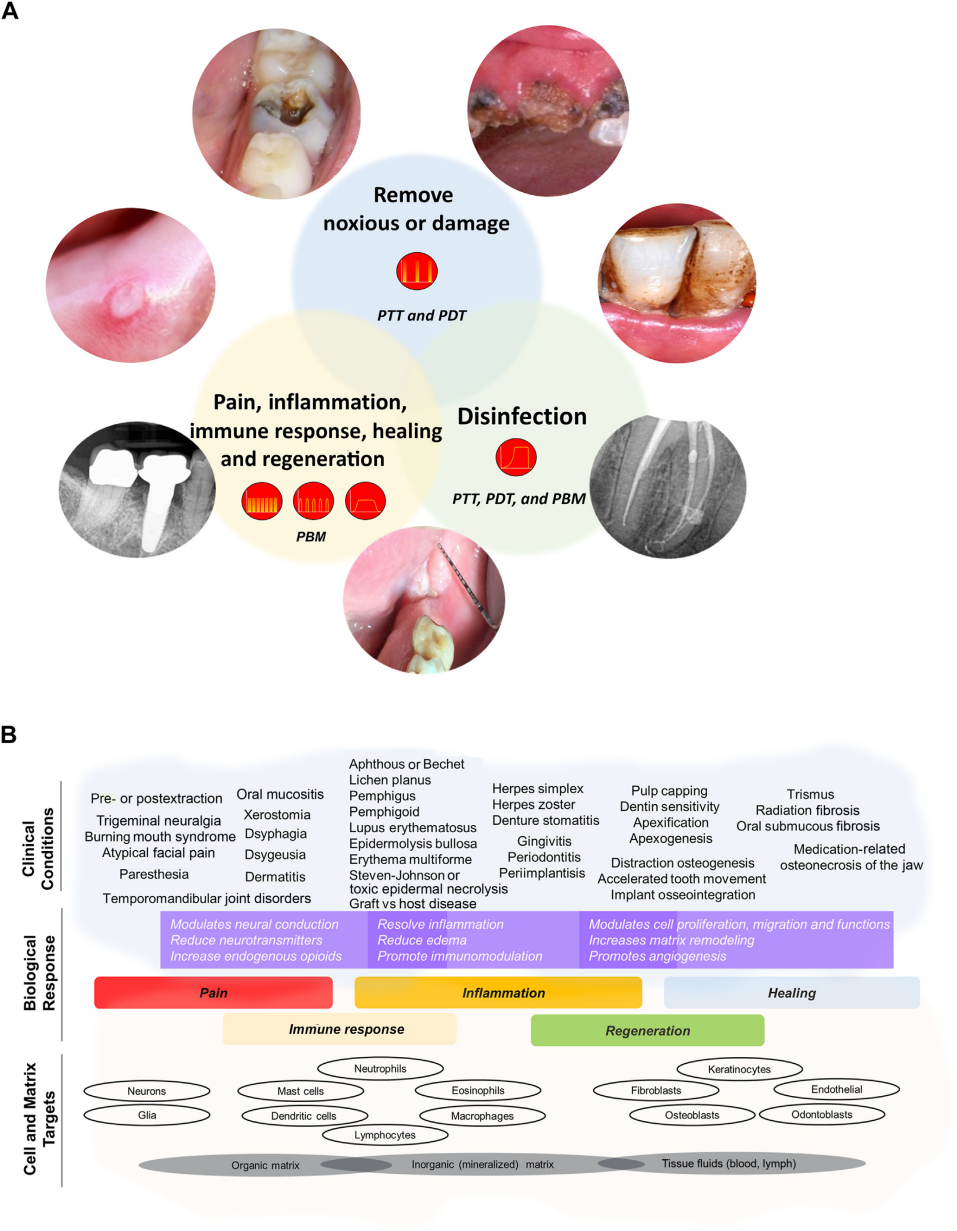
**A.** Outline of various light treatments used in the management of oral and dental diseases. Suitable dose and delivery modifications are necessary to encompass comprehensive treatments that are often synergistic or combinatorial. **B.** The evidence from clinical applications of photobiomodulation (PBM) treatments is outlined. The use of PBM in these scenarios is premised on its ability to evoke therapeutic biological responses, namely alleviation of pain or inflammation, immunomodulation, and promoting tissue healing and regeneration. Laboratory studies have outlined that individual cell lineage responses are effectively modulated by PBM treatments, suggesting future mechanistic studies could focus on their precise pathophysiological roles. PTT: Photothermal therapy. PDT: Photodynamic therapy.

The broad scope of PBM therapeutic benefits extends from its ability to address fundamental biological responses inherent to disease pathophysiology, such as pain, inflammation, aberrant immune responses, and lack of tissue healing and regeneration (Figure 2A).^37^, ^38^, ^39^, ^40^ This rationale mirrors clinical management approaches that rely on analgesics and anti-inflammatory agents, antimicrobial therapy, and immunomodulators to treat oral diseases. A brief list of oral and dental diseases that have been effectively treated with PBM indicates the scope of this intervention in mitigating a broad spectrum of ailments (Figure 2B). As mentioned earlier, PBM treatment in supportive cancer care has reached the pinnacle of clinical evidence from systematic reviews and meta-analyses that have led to clinical practice guidelines for oral mucositis management.^41^, ^42^, ^43^ However, claims for PBM as a panacea for all oral diseases are rather ingenuous, and its therapeutic benefits have been exaggerated primarily because of the lack of a biological rationale for its clinical use.^44^ Many of the PBM applications listed below should be interpreted carefully because of the prevalent positive publication bias and their use as an adjunct or combinatorial with routine care that observes empirical benefits in clinical reports and laboratory studies.

### PBM to mitigate pain and inflammation

The ability of PBM as a noninvasive, nonpharmacological approach to reduce pain and alleviate inflammation has been well documented.^45^, ^46^, ^47^ A key attribute in these studies that makes PBM most attractive for clinical dental application is its efficacy in mitigating pain and inflammation from a broad spectrum of inciting stimuli. The ability of PBM to modulate pain receptors, such as transient receptor potential vanilloid 1, type 1 metabotropic glutamate receptors, tubulin networks, mitochondrial activity, and inhibition of C-X-C motif chemokine ligand 10 expression, has been observed.^48^, ^49^, ^50^, ^51^, ^52^, ^53^, ^54^ These responses are transient and reversible, with no persistent paresthesia or long-term side effects when PBM treatments are performed with appropriate parameters. This includes PBM to reduce preoperative anesthetics and postoperative analgesics by increasing comfort during surgical and tooth extractions, especially impacted third-molar procedures, by reducing edema and swelling.^55^, ^56^, ^57^, ^58^, ^59^ PBM has been reported to be effective in alleviating various facial pain conditions such as trigeminal neuralgia, burning mouth syndrome, atypical facial pain, and other neuropathic syndromes.^60^, ^61^, ^62^, ^63^, ^64^, ^65^ There are several reports on the efficacy of PBM treatments for postsurgical paresthesia and healing, including orthognathic and maxillofacial trauma fixation.^66^, ^67^, ^68^, ^69^, ^70^, ^71^ As noted previously, the most substantial evidence for PBM analgesic efficacy has been indicated in oncotherapy-associated mucositis and other complications such as xerostomia, dysphagia, dysgeusia, and dermatitis, among others.^41^, ^42^, ^43^^,^^72^ Despite the heterogeneity of the underlying pathology in temporomandibular joint disorders from the facia, muscular, osteogenic, or neuropathic pathologies, PBM treatments have been reported to provide symptomatic relief. Hence, they are considered helpful adjuncts in comprehensive management strategies.^73^, ^74^, ^75^, ^76^, ^77^

### PBM modulates the immune responses

The dysregulated or misdirected host immune response results in a chronic aggravated inflammatory response leading to several mucosal and dermal diseases.^78^, ^79^, ^80^, ^81^ PBM treatments have been observed to be capable of mitigating clinical manifestations in aphthous ulcers and Bechet syndrome, lichen planus, pemphigoid, pemphigus, erythema multiforme, epidermolysis bullosa, lupus erythematosus, graft vs host disease, Stevens-Johnson syndrome, and toxic epidermolysis necrosis.^7^ Most of these reports indicate PBM alleviates clinical symptomology and is often combined empirically as adjunctive procedures with routine clinical care. The ability of PBM to modulate both acute and chronic immune cell lineages, such as neutrophils, eosinophils, mast cells, lymphocytes, macrophages, and dendritic cells, has been documented in laboratory and human studies.^82^, ^83^, ^84^, ^85^, ^86^, ^87^, ^88^, ^89^, ^90^, ^91^, ^92^, ^93^, ^94^, ^95^, ^96^, ^97^, ^98^, ^99^, ^100^, ^101^, ^102^, ^103^, ^104^ Furthermore, PBM has been observed to modulate immune cell functions such as proliferation, migration, phagocytosis, and antibody secretion, as well as promote resolution (M1 vs M2 macrophage polarization, promotes interleukin-10 induced forkhead box protein 3 regulatory T cells) and improve immune surveillance response.^88^^,^^105^, ^106^, ^107^, ^108^, ^109^, ^110^, ^111^ There is no evidence for using PBM in oral-premalignant lesions such as leukoplakia and erythroplakia, despite the term laser therapy used in the literature that refers to surgical ablation or PDT instead.^112^, ^113^, ^114^, ^115^, ^116^, ^117^, ^118^ The use of PBM for antimicrobial applications has been based on reducing clinical symptoms in herpes simplex, herpes zoster, denture stomatitis, gingivitis, periodontitis, periimplantitis, and osteomyelitis.^7^^,^^119^, ^120^, ^121^, ^122^, ^123^, ^124^, ^125^, ^126^, ^127^, ^128^, ^129^, ^130^, ^131^, ^132^, ^133^, ^134^, ^135^, ^136^, ^137^, ^138^ The primary biological response evoked by PBM is not directed at the infectious agents but is instead aimed at shoring up the host immune responses to generate antimicrobial peptides, such as human β defensin-2.^8^ Several of these reports reiterate the use of conventional or other forms of laser treatments in conjunction with PBM for optimal clinical therapeutic outcomes.

### PBM treatments to promote tissue healing and regeneration

Resolving pain, inflammation, and immune responses with PBM treatments would inherently promote wound healing and thus lend itself to therapeutic outcomes in several oral diseases. However, direct promotion of the tissue healing responses has been observed with PBM in a cell lineage-directed manner by evoking therapeutic responses in keratinocytes, fibroblasts, endothelial cells, odontoblasts, and osteoblasts.^134^^,^^139^, ^140^, ^141^, ^142^, ^143^, ^144^, ^145^, ^146^, ^147^, ^148^, ^149^, ^150^, ^151^, ^152^, ^153^, ^154^, ^155^, ^156^, ^157^, ^158^, ^159^, ^160^, ^161^, ^162^, ^163^ Some works have placed special emphasis on the role of PBM in stem cells from various anatomic niches that are particularly relevant to oral and craniofacial structures such as dental pulp stem cells, periodontal ligament stem cells, periapical and bone marrow mesenchymal stem cells.^158^^,^^164^, ^165^, ^166^, ^167^, ^168^, ^169^, ^170^, ^171^, ^172^, ^173^, ^174^, ^175^, ^176^ PBM has been used to promote odontogenic repair in pulp capping, tooth desensitization, promoting apexification and apexogenesis posttrauma.^177^, ^178^, ^179^, ^180^, ^181^, ^182^, ^183^, ^184^, ^185^, ^186^, ^187^, ^188^, ^189^, ^190^, ^191^ Several reports on tooth desensitization use various agents (predominantly fluorides) with nonablative photonic doses that do not typically qualify as PBM. There have been reports on PBM promoting periodontal reattachment by regenerating the periodontal ligament and alveolar bone proper.^134^^,^^163^^,^^192^^,^^193^ These PBM healing responses have also been observed in gingival and bone healing, alveolar ridge preservation, distraction osteogenesis, implant osseointegration, and accelerated orthodontic tooth movements.^71^^,^^140^^,^^194^, ^195^, ^196^, ^197^, ^198^, ^199^, ^200^, ^201^

The ideal biological sequelae of wound healing are the restoration of developmental form and function. In contrast, healing responses that go awry result in excessive or misdirected tissue responses, such as necrosis and fibrosis. PBM has been clinically effective in several scenarios, such as postradiation fibrosis and trismus, oral submucosal fibrosis, and medication-related osteonecrosis of the jaw.^68^^,^^202^, ^203^, ^204^, ^205^, ^206^, ^207^, ^208^, ^209^, ^210^, ^211^, ^212^, ^213^, ^214^ The biological rationale for PBM in these contexts has focused on inducing tissue remodeling, resolution of protracted chronic pathobiology, and promotion of resolution and consolidation.^215^^,^^216^ The precise protocol of PBM treatment parameters has varied widely and requires further investigation.^217^, ^218^, ^219^, ^220^

## PBM Mechanisms and Dosing

A major barrier to the mainstream acceptance of PBM therapy has often been its inconsistent clinical benefits. Although this lack of clinical reproducibility highlights the broader biomedical community, especially oncology, the relatively innocuous nature of low-dose light treatments has drawn much more skepticism.^78^ The search for a PBM mechanism has received much attention with the description of the direct absorption of light by cytochrome C oxidase in the mitochondria from the seminal work by Kaaru and Whelan.^221^^,^^222^ The effects on the mitochondrial electron transport chain not only made biochemical sense but also turned out to be an attractive layperson’s explanation as an avenue to transfer physical light energy into an energy-centered cell organelle, the mitochondria. It has since become apparent that PBM evokes beneficial responses in cells that do not have mitochondria (such as erythrocytes and platelets) as well as in cells with dysfunctional mitochondria (mutations).^223^, ^224^, ^225^ Moreover, a biochemical study with mitochondria in isolation using rigorous methodology was unable to replicate the initial biochemical changes previously reported.^226^

**Figure 3:**
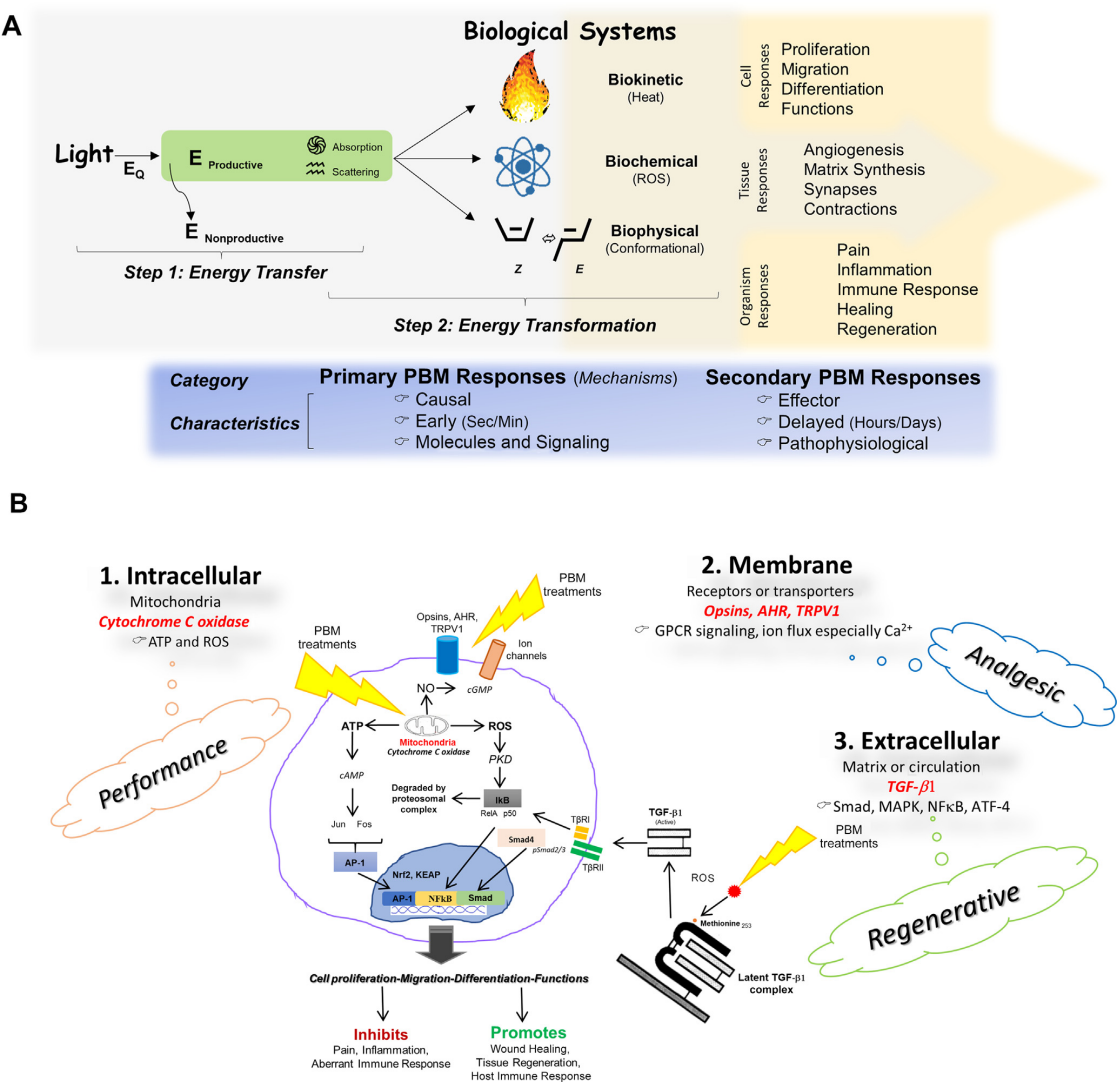
**A.** Understanding of the light-biological tissue interactions in photobiomodulation (PBM) has begun to outline a sequential energy transfer and transformation phenomenon. The eventual cell, tissue, and organ responses are integrated for eventual therapeutic benefits. A distinction from primary, causal responses that provide insights into PBM mechanisms should be distinguished from secondary effector responses. *Modified with permission of John Wiley & Sons, Inc from Young et al.*^229^**B.** The direct PBM mechanisms that have been reported are broadly categorized on the basis of their anatomic location as intracellular, membrane, or extracellular. Given the all-pervasive nature of light-biological interactions, it remains to be investigated if there is significant synergy or antagonism among these individual mechanisms that enable the ultimate therapeutic clinical outcomes. AHR: Aryl hydrocarbon receptor. AP-1: Activator protein-1. ATF-4: Activating transcription factor 4. ATP: Adenosine triphosphate. Ca^2+^: Calcium ion. cAMP: Cyclic adenosine monophosphate. cGMP: Cyclic guanosine monophosphate. E: Energy. E_Q_: Energy_Quantum_. GPCR: G-coupled protein receptor. IkB: Inhibitory κB. KEAP: Kelch-like erythroid-derived cap’n’collar homolog-associated protein 1. MAPK: Mitogen associated protein kinase. NFκB: Nuclear factor κ-B. NO: Nitric oxide. Nrf2: Nuclear factor erythroid 2-related factor 2. PKD: Protein kinase D. pSMAD2/3: Phospho-small mothers against decapentaplegic. ROS: Reactive oxygen species. Smad: Small mothers against decapentaplegic. TβRI: Transforming growth factor-β type I receptor. TβRII: Transforming growth factor-β type II receptor. TGF-β1: Transforming growth factor β1. TRPV1: Transient receptor potential vanilloid 1. z: Enantiomer or chiral conformation.

A review article noted that several articles have claimed that mechanistic PBM insights focus on delayed effector responses.^5^^,^^227^, ^228^ For example, the ability of PBM treatments to improve wound healing involves concerted downstream responses inherent to the biological healing process. These have been inaccurately construed as primary mechanisms because they do not meet the necessary and sufficient conditions for causality. Another key attribute of PBM mechanisms is the timeframe of change that most biologists and clinicians (except the vision and phototransduction community) are not familiar with. These primary mechanisms occur within subsecond ranges and involve biophysical, biokinetic, or biochemical changes involving photon interactions with biological targets (Figure 3A). The interplay among these responses remains an frontier in mechanistic studies.

Investigations have outlined 3 major cellular sites for light-biological target interactions. First, the previously mentioned intracellular PBM targets involving the mitochondria have been well documented. The absorption of light by cytochrome C oxidase results in increased ROS, adenosine triphosphate, and dissociation of nitric oxide in specific pathophysiological scenarios (Figure 3B).^221^^,^^222^^,^^230^, ^231^, ^232^ The improved cell functions resulting from the transiently elevated level induce a concerted gene expression involving nuclear factor erythroid 2-related factor 2 Kelch-like epicholorohydrin-associated protein, mitogen-activated protein kinases, and nuclear factor κB, among others, that are integrated into a therapeutic response. A second PBM mechanism involves the classical elucidation of photoreceptive ion transporters and cell surface receptors, such as nonvisual opsins 2 through 4, transient receptor potential vanilloid 1, Aryl hydrocarbon receptor, purinergic receptors (P2X7, P2Y), and transient receptor potential melastatin-4, 6, and 7.^233^, ^234^, ^235^, ^236^, ^237^ Modulation of these targets by PBM results in potent downstream effects on signal transduction and cellular responses. The third PBM mechanism involves direct activation of a latent growth factor complex, transforming growth factor β-1.^238^^,^^239^ This mechanism has been shown to mediate tissue healing and regenerative responses by promoting the directed differentiation of stem cells. Given the central role of transforming growth factor β in a broad range of human diseases, activation of this pathway could contribute to a better understanding of PBM therapeutic responses in several key disease processes.^78^

## PBM Dosing: A Novel Conceptual Framework

An advancement in the investigation of PBM mechanisms has been the realization that several popular wavelengths, especially 810 nm, with clearly shown clinical benefits, have minimal biological absorption.^240^ Thus, the field has begun to examine the roles of nonelastic scattering and nonlinear effects in PBM responses. These concepts are not unique to PBM, as nonlinear dynamics have been observed in several other biological processes, such as photosynthesis, olfaction, and geomagnetism, considered within an emerging field called quantum biology.^241^, ^242^, ^243^, ^244^, ^245^ PBM represents a low-dose light-biological tissue response field that would logically benefit from these principles and techniques.^246^^,^^247^ The complexity of the quantum, nonlinear effects of light-biological tissue interactions reflects a major paradox with the ease of performing PBM clinical treatments. The simplicity of performing PBM therapy by aiming the device toward the subject and treating it reflects a popular point-and-shoot treatment philosophy.^248^ Although this approach has engendered the ease of PBM clinical adoption and marketing of these devices, it has also resulted in inconsistent, nonreproducible clinical outcomes, undermining the reputation of PBM therapy. The lack of a sound treatment-induced biological response has been a challenge to claims made using specific PBM treatment parameters. However, the cynicism about the therapeutic benefits of PBM appears to ignore the increasing evidence of the clinical therapeutic benefits in humans and animals.^249^ Animal studies are arguably less influenced by placebo responses in chronic, intractable diseases such as Parkinson disease, chronic wounds, Alzheimer disease, and multiple sclerosis.

**Figure 4:**
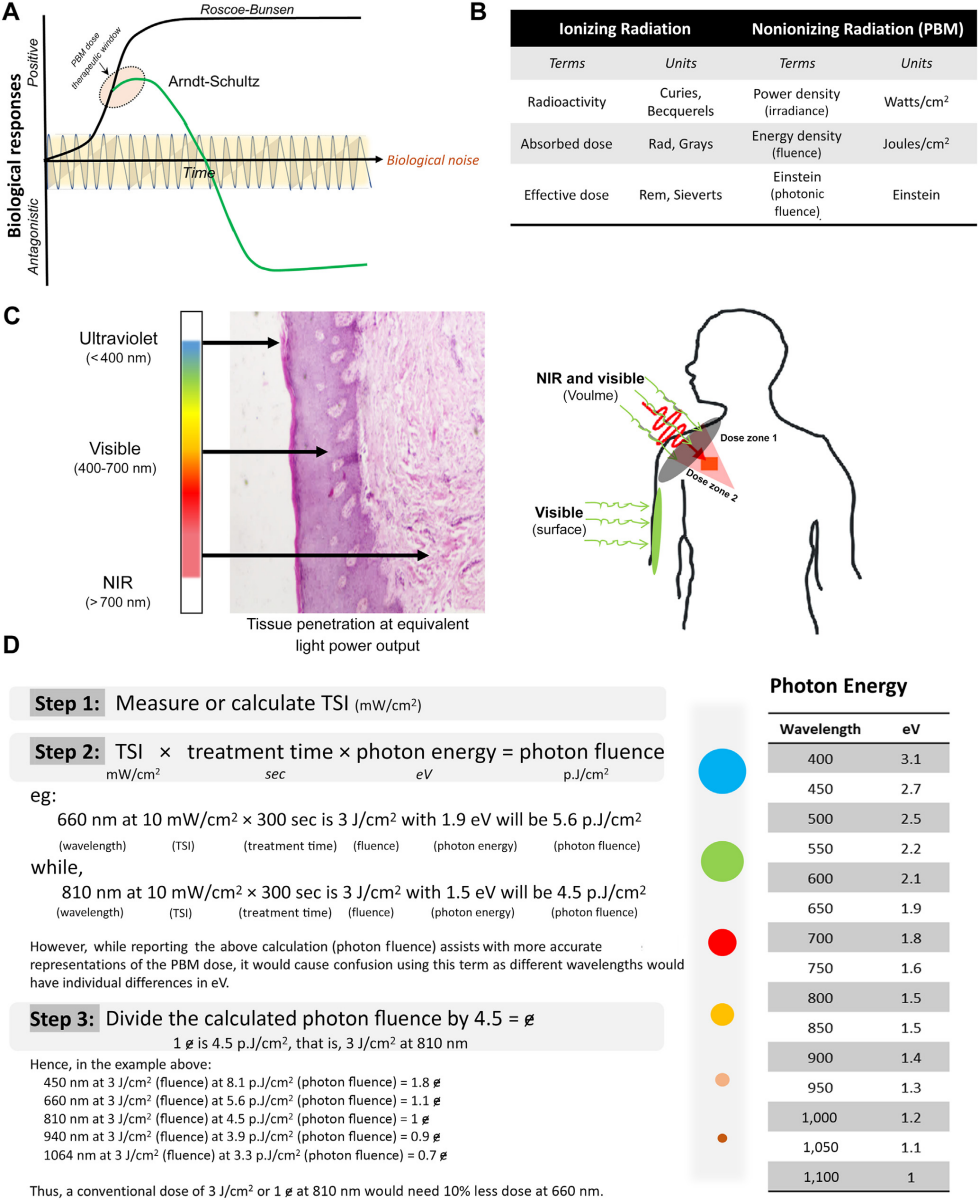
**A.** The biphasic dose evident in photobiomodulation (PBM) responses contradicts the linear, saturation dose models of classic photobiological responses. The nonresponsiveness at the lower dose needs to overcome natural variations during the biological process. The nature of the latter PBM dose responses is antagonistic rather than nonresponsive (saturated) in linear photobiological responses. **B.** Differences between the ionizing and nonionizing PBM physical dose characteristics and units used to describe the physical, biological, and effective clinical responses. **C.** Light penetration is a function of the individual wavelength photon energy, blue being most superficial with near-infrared (NIR) penetrating most deeply. The choice of wavelength is often used to focus the energy in a specific anatomic plane. However, attempts at combining 2 discrete wavelengths have increasingly shown improved clinical efficacy based on energy distributions superficially (both wavelengths; dose zone 1) and deeper tissues (dose zone 2). Zone 1 receives a cumulative dose from both the visible and transiting NIR wavelength. Furthermore, it is conceivable that specific disease states have enhanced or specific chromophores that could be targeted such as increased hemoglobin in an erythematous tissue. **D.** The availability and combinatorial use of various PBM wavelengths have emphasized the need for a universal dose. The inclusion of individual wavelength photon energy (table on the right) enables improved precision and cumulative dose estimations as outlined in the 3-step dose calculation for photon fluence and einstein (ɇ) (810 nm, 3 J/cm^2^ or 4.5 p.J/cm^2^). This dose approach enables improved dose reporting and ease of dose calculations or conversion with various PBM devices, thus enhancing interoperability. TSI: Tissue surface irradiance.

Debate regarding the optimal PBM dose remains a central issue in this field. Given the nonthermal and nondestructive nature of PBM treatments, there has been little rigor in prescription. The ease of performing this therapy, especially in inexperienced or new operators, results in inadvertent overtreatments in their efforts to maximize benefits. This is further compounded by the simplicity of performing this treatment, which is promoted by commercial marketing. The conceptual framework of PBM dosing follows the Arndt-Schultz or Hormesis dose curve, first described in toxicology in 1888 based on the stimulatory effect of low-dose toxins.^250^ Improved understanding of the nature of the biphasic and triphasic biochemical responses has further helped elaborate the J- or U-shaped dose curves in which low-dose stimulation and high-dose inhibition are observed (Figure 4A). The positive therapeutic responses at these low doses have been compared with physiological preconditioning or eustress.^250^ These therapeutic effects on reducing pain or inflammation require somewhat higher PBM doses, albeit within the nonthermal regimen, often accomplished by pulsing or moving the PBM light source. Furthermore, several classical, linear photobiological responses, such as the Grothus-Draper, Stark-Einstein, and Roscoe-Bunsen laws, are not explicitly evident in PBM responses. Although light is a discrete part of the electromagnetic radiation spectrum, the linear nonthreshold dose model routinely used for ionizing radiation does not appear to be valid for nonionizing PBM wavelengths (Figure 4B). This also implies that discrete clinical and laboratory safety guidelines must be instituted for the safe use of lasers or light devices for PBM, as elaborated in a subsequent section.

### From theory to clinical PBM practice

Two practical aspects of clinical PBM dosing require attention and are discussed in more detail in other articles that have been briefly summarized in a review article.^5^^,^^251^, ^252^, ^253^, ^254^, ^255^, ^256^, ^257^, ^258^, ^259^, ^260^, ^261^, ^262^, ^263^, ^264^, ^265^, ^266^, ^267^, ^268^, ^269^ First, the PBM device parameters such as choice of wavelength or combinations (with multiple wavelength units; Figure 4C), PBM light sources (laser vs light-emitting diodes [LEDs]), source-specific beam attributes (spectral range, half-width at full maximum, polarization), mode of operation (continuous wave vs pulsing), irradiance (power density mW/cm^2^), fluence (energy density J/cm^2^), and treatment time (session and repetitions). A dose concept has suggested accounting for the individual wavelength photonic energy (eV) that focuses on multiple modes of energy transfer, including absorption and inelastic scattering.^229^ The importance of scattering is evident within tissues and is influenced by the wavelength and beam diameter (spot size), which are inversely correlated with absorption. The photon fluence (p. J/cm^2^) is calculated by including the individual wavelength-specific photon energy to the irradiance and treatment time or fluence (J/cm^2^) (Figure 4D). As the photon fluence makes the generalized dosing recommendation more complex, 810 nm at the popular 3 J/cm^2^, resulting in 4.5 p.J/cm^2^, has been used as 1 einstein.^229^ The choice of 810 nm as a standard reflects the median energy in the PBM wavelength range (400-1,100 nm), minimal (water) absorption among known biological chromophores, and widespread use (besides 660 nm) in the PBM literature. The new dose paradigm effectively communicates the PBM dose and enables universal harmonized dosing irrespective of the device and wavelengths available globally. Accurate reporting and interconvertibility of widespread PBM use with this new dose concept are attractive attributes. Moreover, the availability of multiple wavelengths and their combinations, as mentioned previously, is premised on their inherent differences in light penetration for treating larger and more uniform tissue volumes. Photon fluence enables cumulative doses to be represented most accurately, and sophisticated dose-planning studies are being pursued.

The second major category of dose variables was the clinical delivery approach. This includes using a single probe vs arrays (LEDs), stationary vs scanning, distance, and spot size. Former focal PBM treatments are usually performed at a specific site of pathology, such as a painful spot, clinically evident lesion (sore or wound), or treatment site. In cases of inflammation, specifically edema or neuropathy, some protocols suggest treating the adjacent draining lymphatics or afferent neurosensory systems to aid in resolution. However, several ailments do not present a precise site for PBM therapy; therefore, a generalized treatment field was used. Thus, there have been suggestions to target explicit anatomic sites based on acupuncture points.^270^, ^271^, ^272^, ^273^ In contrast to these targeted approaches, evidence from controlled animal studies suggests that nontargeted, generalized PBM treatments have prominent systemic benefits.^274^, ^275^, ^276^ This global PBM approach has gained credence with a growing number of human clinical studies using whole-body PBM treatments, reinforcing the use of nontargeted dose delivery.^277^, ^278^, ^279^ Ongoing studies have examined the use of either approach in specific disease contexts. It is foreseeable that local, global, or synergistic combinations of both approaches will be used in future protocols. There is interest in precise dosimetry with external light sources for PBM delivery to deep-seated targets such as the inner lining of the oral cavity, temporomandibular joints, or trigeminal ganglia. Inspired by radiation therapy and PDT approaches, sophisticated dosimetry techniques, such as Monte Carlo simulations and neural networks, have been attempted.^16^^,^^18^^,^^19^^,^^280^, ^281^, ^282^, ^283^, ^284^, ^285^, ^286^, ^287^ However, unlike PDT and radiation treatments, in which the mean lethal light dose is documented, the effects of the PBM dose on specific target lineages for directed therapeutic responses remain to be fully understood.

Another report outlined the integrated concept of cumulatively accounting for both distance and spot size, termed tissue surface irradiance (TSI) (mW/cm^2^), which increases PBM reproducibility.^288^ This approach is practical in clinical scenarios and conveniently assessable in controlled laboratory studies. It is particularly helpful with point sources, multiple probes, and large array units to rigorously determine the effective power density and reproducibility of the PBM treatment. Moreover, TSI allows accurate estimation of dose delivery with any probe movements (raster, circular, stamping), modes that are often used to ensure a nonthermal regimen is maintained throughout PBM treatments.^21^ Generally, lower TSI (0.1-25 mW/cm^2^ in vitro and in vivo) appear to be optimal for wound healing-tissue regenerative applications, while higher TSI (up to 150 mW/cm^2^ in vivo) appear to be most effective at relieving pain and inflammation. Large arrays also use higher irradiance; however, care must be taken so that no prominent tissue heating (sauna effects) or patient discomfort is perceived. Another practical concern in clinical dentistry is the line of sight of PBM treatments with limited oral access. Several practical techniques, such as scanning modes and digital sensors, have been used to overcome this issue.

## Clinical Considerations for PBM Therapy

Prior studies have reported the discrete use of PTT and PDT vs PBM. The first 2 techniques eventually result in reduced pain, inflammation, and tissue healing.^289^, ^290^ However, these are consequences of the primary therapeutic intervention, which is the removal of the source of the pathology. However, PBM directly stimulates the host’s therapeutic responses, either alleviating pain or inflammation and promoting tissue healing and regeneration. Appropriate clinical diagnosis and a rational choice of therapy should drive the clinical use of these individual forms of light treatment. There are numerous reports on the growing number of PBM applications that can be incorporated into a large portion of clinical dentistry, such as promoting tissue reliance and healing in oncotherapy-associated oral mucositis, pain relief after tooth extraction or inflammation, and mitigating autoimmune lesions. A partial list of ailments is provided here with the level of evidence for PBM efficacy; the reader is referred to more comprehensive reviews of the literature for individual protocols.^7^ Many documented case reports and conference reports remain anecdotal and are arbitrarily rationalized, making assessment of true PBM efficacy difficult, if at all feasible, in several clinical scenarios. Nonetheless, PBM has clear use as an adjuvant in several clinical scenarios, whereas it could be the primary intervention in other clinical conditions that require further careful and rationalized investigations.

Dentistry is poised to lead the clinical translation of PBM treatments based on evidence from multiple systematic reviews and meta-analyses performed by the Multinational Association for Supportive Care in Cancer and the International Society for Oral Oncology.^41^, ^42^, ^43^ They recommended the routine use of PBM in managing oncotherapy-associated oral mucositis.^41^, ^42^, ^43^ This analysis observed a significant reduction in the pain, incidence, and severity of oral mucositis. However, this report also noted considerable variance in the PBM device parameters and the manner of use in these successful clinical protocols, precluding a universal PBM protocol recommendation.^43^ A follow-up assessment using WALT attempted to address these discrepancies by outlining a broad range of treatment parameters based on the understanding of PBM responses.

### PBM Clinical Safety

**Figure 5:**
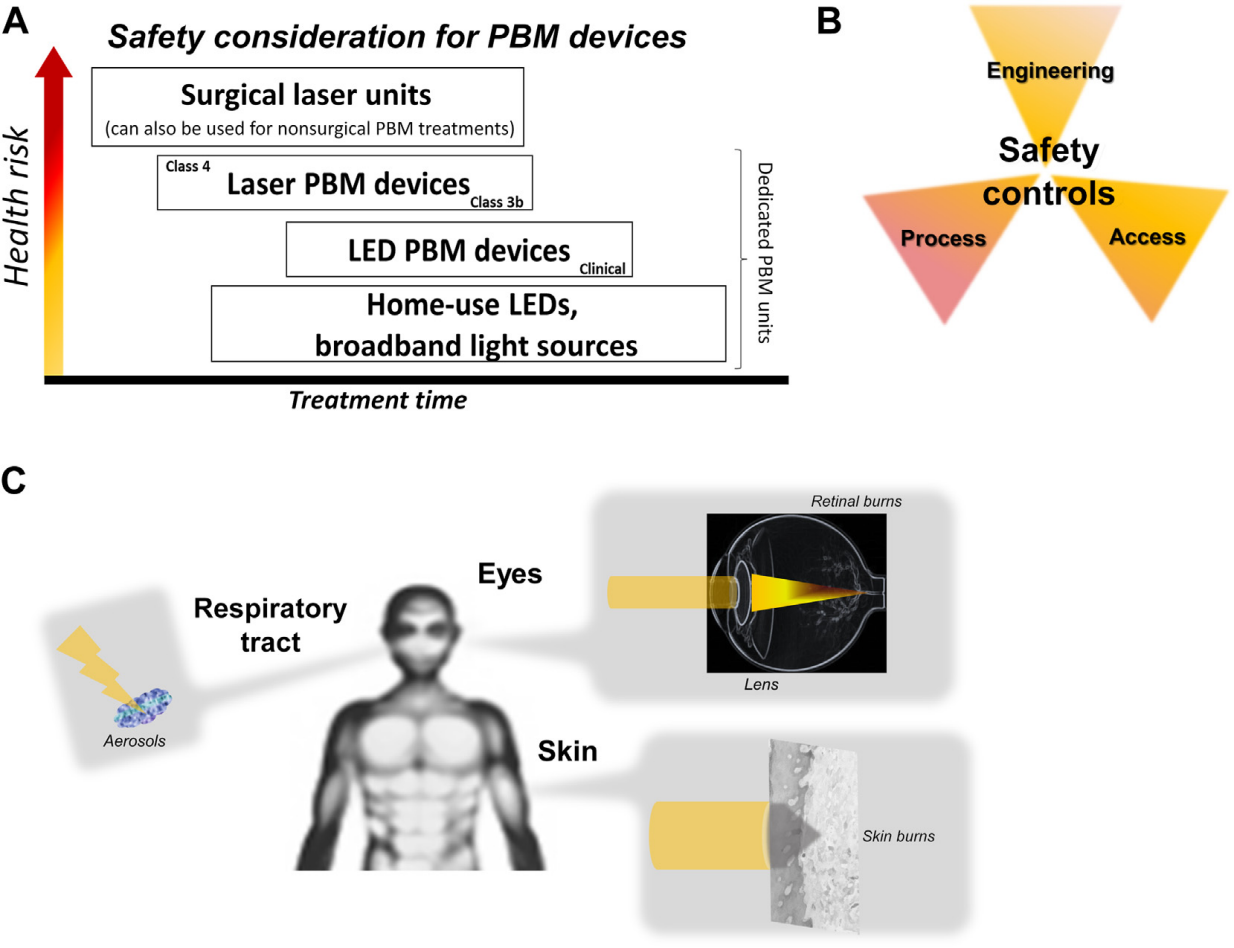
**A.** There is a wide range of clinical safety requirements and potential health risks from the broad range of photobiomodulation (PBM) devices. These range from the use of low-dose light-emitting diodes (LEDs) for home use to the adaption of surgical lasers with diffusers that reflect the highest potential for tissue damage. Despite inherent differences between LEDs and lasers used for PBM, clinical use must adhere to the highest health regulatory policies to ensure safe clinical operations. **B.** The clinical safety measures can be broadly categorized into 3 discrete aspects namely access, engineering, and process controls or safeguards. Each category must have explicit guidelines and documentation for safe PBM device use. **C.** The 3 major organs that are at risk with light devices are the eye, skin, and respiratory tract. Stringent procedural protocols and personal protective equipment (masks, eyewear) are critical to ensure safe and effective clinical PBM treatments.

There is little theoretical evidence or clinical reporting of any adverse events associated with PBM.^280^^,^^291^ This is primarily attributed to the lack of direct phototoxicity of the minimal linear energy transfer of visible and near-infrared nonionizing PBM wavelengths. Furthermore, there was minimal indirect phototoxicity at low doses of the generated redox reagent. The clinical safety of PBM has been well documented, and the few reports on adverse events have been primarily ascribed to the improper use of laser PBM devices.^21^, ^292^, ^293^, ^294^ Guidelines for the safe use of these light devices in health care settings are outlined in the American National Standard for Lasers (Z136.1), American National Standard for Safe Use of Lasers in Health Care (Z136.3), and International Electrochemical Commission (62471) for LED use.^295^, ^296^ These documents outline the 3 broad categories of engineering, access, and process control necessary for the safe clinical use of laser and LED PBM devices (Figure 5A). Conventionally, the 2 organ systems in which PBM safety is the most concerning are the eye and skin (including the mucosa), which are exposed to direct or specular reflections (Figure 5B). Emphasis on eye safety is paramount because of the inherent light responsiveness and amplification of incident light via the eye lens. Protective eyewear with appropriate wavelength-specific optical density for a specified wavelength is critical. Although no significant laser-generated aerosols were anticipated with class 4 lasers at low PBM doses, masking to protect the respiratory tract is advisable.

Another area of PBM safety concern is its use in oncotherapy-associated oral mucositis, in which treatments are performed in patients with active tumors, especially in those with head and neck cancers. Laboratory and clinical research studies have examined the safety of PBM in tumor-bearing areas and found no evidence of the proliferation of tumors or pretransformed cells.^297^^,^^298^ Retrospective human clinical studies have further validated PBM safety, with no increase in recurrence or second primary or secondary tumors.^299^^,^^300^ These reports suggest that indirect PBM benefits may be based on the timely completion of prescribed oncotherapy regimens without interruptions from treatment-associated complications. Observations of synergistic combinatorial effects with oncotherapy promoting the apoptosis of tumor cells after PBM remain to be validated using rigorous methodologies.^301^

## Contraindications for PBM Treatments

No direct contraindications to PBM have been reported. Nonetheless, direct treatment of the fetus in a pregnant person or an apparent tumor mass should be avoided. It is increasingly apparent that both populations would benefit from PBM therapy and hence do not reflect absolute clinical contraindications. There have been some concerns regarding the use of pulsed laser beams for PBM in patients with a history of seizures, but no documented adverse events have been reported. Another anecdotal statement is the avoidance of PBM in patients with hyperthyroidism, which remains unknown. There are a few clinical situations in which the PBM dose can be adjusted (reduced) based on the patient’s response. The first scenario involves patients with red hair carrying melanocortin-1 receptor mutations, who are known to have increased sensitivity to sunlight. Second, patients take specific classes of drugs (eg, tetracycline) or natural products (eg, St. John’s Wort) that transiently increase their photosensitivity. A medical history of all medications and supplements must be documented. Finally, there is a rare small group of patients with increased genetic susceptibility to skin photosensitivity, such as patients with xeroderma pigmentosum, Cockayne syndrome, and Blooms syndrome.^302^, ^303^, ^304^

## Emerging Issues and Future Directions for PBM

### Consent and documentation

Health care providers of PBM must explain to the patient and the risks, benefits, and alternatives to treatment and obtain appropriate informed consent. Documentation of the procedural PBM parameters is required to improve the rigor and reproducibility of future treatments (Appendix). Besides regulatory compliance, the clinician and PBM fields would benefit immensely from careful documentation for the future assessment of clinical efficacy, safety, reimbursements, clinical care guidelines, cost, and quality of care metrics.

### Broad implications of PBM on health and wellness

A challenge with this treatment is the breadth of its scope of clinical applications. This has been attributed to its impact on fundamental pathophysiological disease responses, such as pain, inflammation, immune responses, and tissue healing. PBM treatments have been used as both primary and adjunct interventions for several human ailments. Several areas of future investigation appear to be critical for the field to advance, including fundamental mechanistic investigations to provide rationalized therapeutic interventions for disease states. This could provide key evidence for partial and nonresponders to PBM therapy. Another prominent area of inquiry is controlled redox signaling, both prooxidant in the early phase and potent antioxidant response in the later phases, as evident in PBM responses. Given the ubiquitous nature of light, there are avenues for practical concepts such as light as a supplement, drug (photoceutical), and for hygiene. The importance of the latter is evident in the use of blue-light filters in digital devices and displays. Another major area for optimal PBM therapy is individual clinical scenarios, each requiring study design and execution that require significant investment in resources and infrastructure, especially via industry-government partnerships.

### Clinic vs home use

PBM treatment with laser devices is routinely provided in a clinical setting. However, the availability of LED PBM devices with strong clinical safety profiles has increased the popularity of at-home applications. This has become particularly attractive in nonurban, remote areas or in those patients with protracted, chronic diseases requiring interventions between clinical visits. Home-use compliance and safety (inadvertent overuse or misuse) are being addressed using sensors and wireless (wireless fidelity, radio frequency identification) connectivity while maintaining data integrity.

### PBM education, training, access, and reimbursement

The clinical diagnosis and treatment planning must form the fundamental basis of prescribed PBM treatment. The ease of use of PBM and its availability, especially LED home-use devices, have raised queries regarding the appropriate training and qualifications of caregivers. Device-specific and advanced clinical training is a key aspect of future PBM education and deserves attention. Regulatory training requirements should balance access to the potential risks of PBM intervention (sFigure). With the growing list of oral and dental diseases showing the clinical efficacy of PBM, especially oncotherapy-associated oral mucositis, the barrier is the lack of medical reimbursement for PBM. Although the clinical evidence of safety and efficacy from 35 placebo-controlled, multicenter human trials in a systematic review and meta-analysis are unequivocal, cost-of-care analysis has also shown significant improvements in quality of life, reducing the fiscal burden and improving health care standards, supporting the inclusion of PBM as a routine part of bundled supportive cancer care.^41^, ^42^, ^43^^,^^299^^,^^305^, ^306^, ^307^

Many other dental issues could also benefit from PBM, such as preoperative and postoperative pain during extractions, aphthous ulcers, and xerostomia. However, these require stringent therapeutic validation studies and quality of care analysis, as per the FDA guidelines for PBM devices.^308^ Owing to the broad scope of possible PBM clinical applications, a suggestion is to implement a time-based procedural reimbursement, as followed for anesthesia, to maximize flexibility for clinical implementation of variation in the duration and repetitions of PBM treatments. The FDA document was the first formal recognition of PBM by a federal agency as a discrete form of light treatment, which is a significant advance from its prior nonheating lamp category. Future iterations of these directives should reflect the recognition of anatomic site and disease pathophysiology-specific PBM devices and their applications, furthering cost-of-care analysis and enabling reimbursement guidelines.

## Conclusions

There is scientific evidence from controlled laboratory studies and human clinical trials regarding the use of PBM therapy in dentistry. Advances in device technology, mechanistic understanding of therapeutic responses, and the development of clinical guidelines have led to the implementation of PBM in clinical dentistry. As with all technologies, further investigations using PBM will be able to refine the parameters and tissue interactions for optimized patient care. This technology must be used with the same rationalized clinical approach that addresses a specific diagnosis with a prescribed treatment regimen. This should be based on a thorough understanding of light-biological tissue interactions, target tissue composition, and directed evoked therapeutic biological responses. Education and training are important for appreciating the benefits and limitations of this technology.

## Disclosure

Dr Arany did not report disclosures.

## Figures and Tables

**sFigure:**
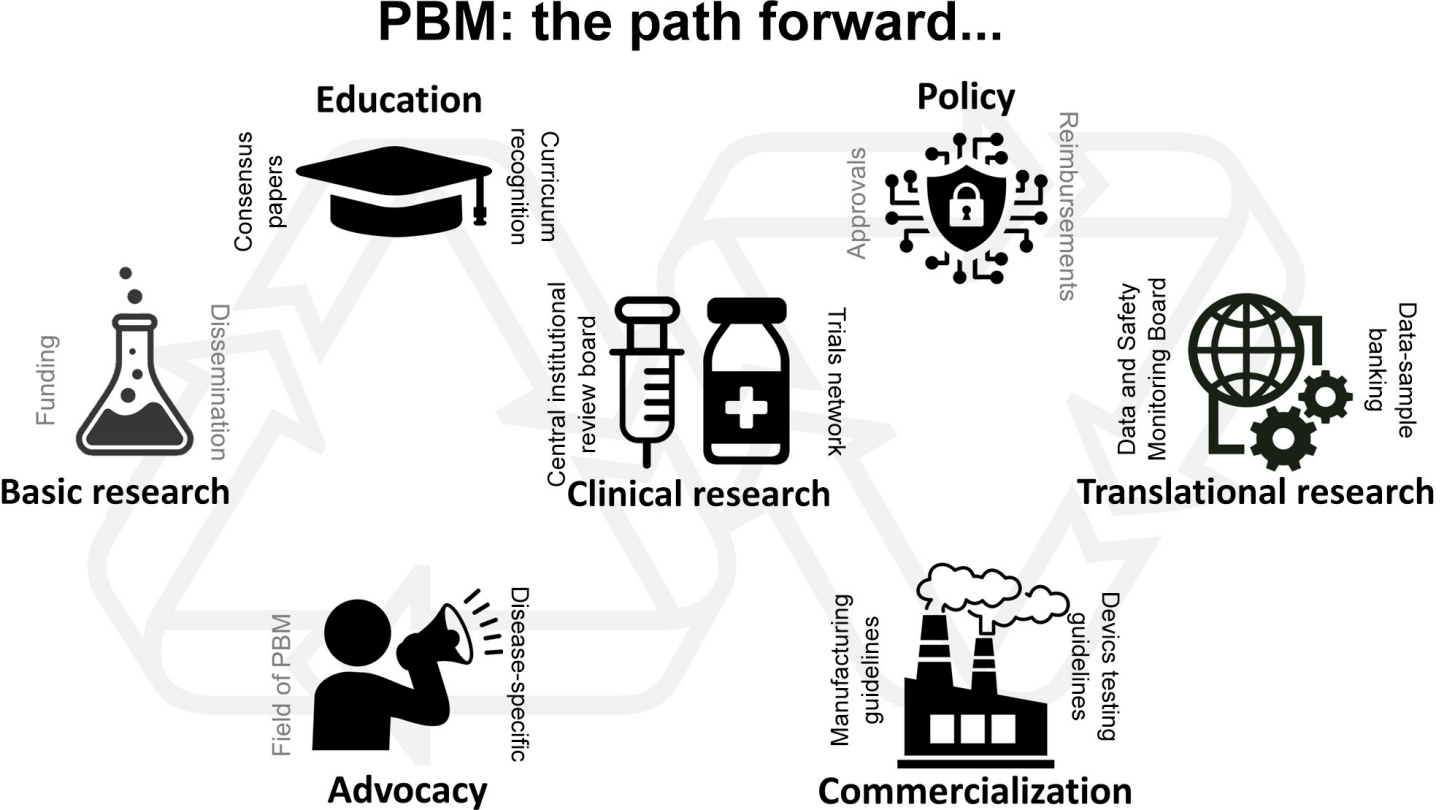
The interplay of various domains of photobiomodulation (PBM) activities, from laboratory-based basic research, clinical trials, development of new devices and protocols, education, patient advocacy, and regulatory policy, are leading to the development and implementation of PBM to improve health and wellness. Icons from websites uxwing.com, IconPNG.com, and flaticon.com.

## Appendix: Recommend Documentation for Photobiomodulation Procedures

Suggested photobiomodulation parameters to be documented for clinical treatments. It is further recommended that standard operative procedures be developed with the specific device at each center for specific diseases or procedures to enable tracking of clinical variance with individual patient treatments.

• Practitioner performing the procedure
  ○ Other individuals in the treatment area or laser-controlled area
• Informed consent
  ○ Risk, benefits, and alternatives explained
  ○ Acceptance documented
• Safety procedures used
  ○ Eye protection with optical density for specific wavelength noted
  ○ Local exhaust ventilation or high-volume evacuation
  ○ Cooling (if used)
• Diagnosis and purpose
• Treatment objective(s)
  ○ Reducing pain
  ○ Managing inflammation
  ○ Modulating the immune response
  ○ Enhancing healing
  ○ Other(s)
    ▪ Please specify: _______________
• Type(s) and model(s) of device(s) used
  ○ Light-emitting diode
  ○ Laser
• Wavelength(s) used (nm)
• Temporal mode(s)
  ○ Continuous wave
  ○ Pulsed
    ▪ Hertz rate
    ▪ Duty cycle
• Manner of use: in-contact or spot size and distance from tissue
• Technique used:
  ○ Stamping
    ▪ Number of sites
  ○ Sweeping motion
    ▪ Speed
• Power used or tissue surface irradiance (mW /cm^2^)
• Location, treatment time per location, and number of treatment(s)
• Patient’s perception and response to treatment
  ○ No notable discomfort, feeling warm during treatment, etc.
• Reduction in discomfort, pain, swelling
• Posttreatment instructions provided: _____ Yes _____ No
• Follow-up treatment scheduled: _____ Yes _____ No
